# Treatment for Major Depressive Disorder by Repetitive Transcranial Magnetic Stimulation in Different Parameters: A Randomized Double-Blinded Controlled Trial

**DOI:** 10.3389/fpsyt.2021.623765

**Published:** 2021-04-06

**Authors:** Tingting Zhang, Yueqin Huang, Yi Jin, Xiaoyan Ma, Zhaorui Liu

**Affiliations:** ^1^National Health Commission Key Laboratory of Mental Health (Peking University), National Clinical Research Center for Mental Disorders (Peking University Sixth Hospital), Peking University Sixth Hospital, Peking University Institute of Mental Health, Beijing, China; ^2^Brain Health Leadership Foundation, Reno, NV, United States

**Keywords:** repetitive transcranial magnetic stimulation, major depressive disorder, randomized double-blind controlled trial, hamilton depression rating scale, left dorsolateral pre-frontal cortex

## Abstract

**Background:** Repetitive transcranial magnetic stimulation (rTMS) has been proven to be safe and effective in treating major depressive disorder (MDD). However, the treatment parameters of rTMS are still divergent and need to be optimized further. The aim of this study was to compare the efficacy of rTMS in treating MDD with different parameters of stimulating frequency and location, and course of treatment.

**Methods:** A total of 221 patients with MDD were recruited in the randomized, double-blind, controlled trial. All eligible patients were randomly assigned into four treatment groups: (1) 10 Hz in left dorsolateral pre-frontal cortex (DLPFC) (*n* = 55), (2) 5 Hz in left DLPFC (*n* = 53), (3) 10 Hz in bilateral DLPFC (*n* = 57), and (4) 5 Hz in bilateral DLPFC (*n* = 56). The patients received treatment for 6 weeks and an additional 6-week optional treatment. The efficacies were evaluated by Hamilton Depression Rating Scale-24 items (HDRS) and Clinical Global Impressions Scale (CGI). The trial is registered at the Chinese Clinical Trial Registry as ChiCTR-TRC-12002248.

**Results:** The ANOVAs of HDRS scores up to 6 weeks and 12 weeks with repeated measure of time showed a significant effect of duration without statistical difference among four treatment groups and no significance when time was interacted with inter-group as well. The response rates up until the 5th week were significantly different with the previous week.

**Conclusions:** It concludes that there were no statistical differences in the efficacy of rTMS between unilateral left and bilateral DLPFC, and between 5 and 10 Hz for treating MDD.

## Introduction

Major depressive disorder (MDD) leads to a big public health concern with a considerably high level of disease burden. A recent epidemiological survey showed that MDD was prevalent in China with a 2.1% 12-month prevalence and a 3.4% lifetime prevalence (1). Repetitive transcranial magnetic stimulation (rTMS) is a non-invasive somatic therapy, which has been proven to be safe and efficacious for treating MDD. It was first introduced as a method for inducing currents into the brain in 1985 by Barker and was used for MDD in 1995 (2). From then on, it has been repeatedly demonstrated to have a therapeutic benefit for MDD in many clinical trials (3–6). Repetitive transcranial magnetic stimulation pulse works on brain function directly by entrainment of cerebral oscillations to the frequency of stimulation and resetting the thalamocortical oscillators (7, 8). Repetitive transcranial magnetic stimulation with 5–20 Hz was considered to increase cortical excitability and might correct the abnormally low level of cortical activity in left dorsolateral pre-frontal cortex (DLPFC), while low-frequency (<1 Hz) rTMS was thought to decrease cortical activity (9, 10). It was proven that high frequency in left DLPFC and low frequency in right DLPFC were both effective for MDD (4, 11–13). In recent years, the bilateral DLPFC has been found to be another successful stimulation site (14, 15). Several meta-analyses have also found moderate to large effect sizes for MDD (16–20). After many systematic clinical trials, the U.S. Food and Drug Administration (FDA) cleared rTMS in 2008 for treating MDD with the protocol of 10 Hz in left DLPFC (21). Among previous studies, many parameters' rTMS were shown to be effective for MDD, which brought about the question of what the mechanism of action really was. Researchers have used magnetic resonance imaging to help locate the site of action and explored the mechanism of different frequencies. The diversity and complexity of the choice of parameters have limited the clinical application. Based on the confirmed effect, it was necessary to test the superiority of different parameters. Several small-sample researches showed that high-frequency rTMS in left DLPFC had an equivalent efficacy to low-frequency rTMS in right DLPFC (22–26). A study conducted by Fitzgerald also found no substantial difference in response between the low-frequency right DLPFC and sequential bilateral DLPFC (15). However, most of the studies above were limited by a small sample size. Duration of treatment is another important parameter for MDD. Most of the rTMS trials were only conducted for 2 weeks, while some prolonged the treatment duration to 4 weeks or longer (7, 27). No studies have been designed to compare the efficacy of rTMS in different durations of treatment. It is thus worth further investigation whether a certain combination of stimulation parameters is superior to others in a head-to-head study.

The first aim of the present study was to test the clinical prominence of combination of four parameters in the treatment of MDD. The study selected two active frequencies (5 vs. 10 Hz) and two locations (left DLPFC vs. bilateral DLPFC). The second aim of the trial was to investigate the efficacy of rTMS in different durations. The study intends to provide clinical guidance for optimal choice of rTMS parameters in MDD treatments, rather than replicate the efficacy test against sham.

## Materials and Methods

### Participants

Between June 2012 and August 2014, 221 eligible patients who met the DSM-IV criteria for MDD by the Structured Clinical Interview for DSM-IV (SCID) from three psychiatric hospitals and one psychiatric department of general hospital were recruited and allocated to four treatment groups of rTMS.

To be eligible for the study, the patients had to be between the ages of 18 and 65 years and to have at least a score of 20 on the Hamilton Depression Rating Scale-24 items (HDRS) at baseline. Patients were required to be free of any antidepressant and benzodiazepine or to have a minimum 2-week washout prior to entering the study under the supervision of psychiatrists. Patients with psychotic symptoms, bipolar disorder, substance abuse, or suicide ideation or attempt were excluded from the study. Patients with pregnancy, history of electroconvulsive therapy, epilepsy, and utilization of a cardiac pacemaker or any intracranial mental implant were also excluded from participation to the study.

Previous research has shown that stimulating left DLPFC produces a response rate of 45% and stimulation of bilateral DLPFC produces a response rate of 20% (11, 28). The difference of response rate between the low and high frequency is larger than the difference between the two locations (29). The sample size therefore was calculated using the response rate of stimulating location in order to account for the smaller differential between these two conditions. According to the sample size formula, letting α = 0.05 and β = 0.20 and using a two-sided test, the estimated sample size was *N* = 54 for each treatment group, resulting in a total of 216 participants.

$$n=\frac{{\left[z_{\alpha }\sqrt{2\bar{p}(1-\bar{p})}+z_{\beta }\sqrt{p_{1}(1-p_{1})+p_{2}(1-p_{2})}\right]}^{2}}{{\left(p_{1}-p_{2}\right)}^{2}}$$

Where *p*_1_ and *p*_2_ were the response rates of two groups, respectively. $\bar{p}$ = (*p*_1_ + *p*_2_)/2.

### Randomization and Masking

After the baseline data and written informed consent for participation were obtained, the patients were assigned randomly into one of the four treatment groups: (1) 10 Hz and left DLPFC with figure-eight coil, (2) 5 Hz and left DLPFC with figure-eight coil, (3) 10 Hz and bilateral DLPFC with round coil, and (4) 5 Hz and bilateral DLPFC with round coil. By design, the round coil is for diffused stimulation where figure-8 is more focus on point. One of the arguments about TMS treatment effect is due to its stimulation on specific anatomic location or just electromagnetic pulses in the brain. These two coil configurations were choices available during the study to test the hypotheses. In the present research, we placed the round coil centered at the mid-pre-frontal cortex including left and right DLPFC. The randomization code of 1–4 was generated by a computer, which was blinded to both the study participants and the clinical evaluators. The participant was assigned a randomization code according to the order of entry and was allocated to the relevant treatment group. The treatment allocation was totally concealed from the recruited participants and evaluators. Although the participants might know the type of the coil, they did not know the efficacy difference between the two coils. Meanwhile, the frequency was masked to the participants.

### Treatment

Treatment stimulation was delivered at an intensity of 100% resting motor threshold (MT). On the initial treatment, the identification of the resting MT of the target stimulation area was performed using a method of limits (30). Resting MT was considered as the minimum TMS intensity sufficient to produce a predefined motor-evoked potential (the right-hand fingers twitching appears visibly) in the contralateral abductor pollicis brevis (APB) in 5 out of 10 trials when the hand was relaxed. The stimulus intervals were 5 s each. The stimulation site was placed 5 cm anterior to the maximal APB site in the parasagittal plane.

The participants received rTMS treatments daily, five sessions a week for 6–12 consecutive weeks using a Magstim Rapid 2 stimulator. Each session lasted 30 min, and each minute included 4 s of active stimulation and 56 s of rest. After 6-week treatment, the patients could choose to continue the trial treatment for an additional 6 weeks.

The ratings were performed by trained evaluators weekly who were masked to treatment arms. The HDRS and Clinical Global Impressions Scale (CGI) were administrated at each evaluation. Clinical evaluators performed a clinical evaluation at baseline and once a week after the patient received treatment. All the authors also had no access to information that could identify participants.

### Outcome Measures

The primary outcome was the change of HDRS scores at 12 weeks of treatment. Secondary outcomes were CGI score and response rate. Response, a binary variable, was defined as a >50% reduction in HDRS score from the baseline [(HDRS baseline – HDRS after treatment)/HDRS baseline × 100%]. Response rate was the rate of the responders in treatment arms.

### Statistical Analysis

SPSS was used for statistical analysis. Continuous variables, such as age and HDRS baseline score were described as mean ± SD. Categorical variables such gender and marital status were reported as frequency. Continuous and categorical variables were compared between groups by the analysis of variance (ANOVA) and Chi-square analysis (Fisher's Exact Test was calculated when needed), respectively. The efficacy of rTMS therapy, measured by HDRS, was assessed using ANOVA with repeated measures across time, among four groups. The intent-to-treat (ITT) sample was defined in the protocol as the patients with baseline assessment and at least one assessment post-baseline. Last Observation Carried Forward (LOCF) method was used to impute the missing data of all continuous outcome variables in the ITT sample for ANOVA with repeated measures. Per-protocol (PP) analysis, which meant only the completed cases in the analysis were included, was applied as well. For the analysis of response rate, the generalized estimating equation (GEE) was used. In GEE, the data set was transformed from case union to time-case union without imputation the missing data. A *P* < 0.05 was considered statistically significant, and all reported *P*-values were two-sided.

## Results

### Characteristics and Deposition of Participants

**Figure 1:**
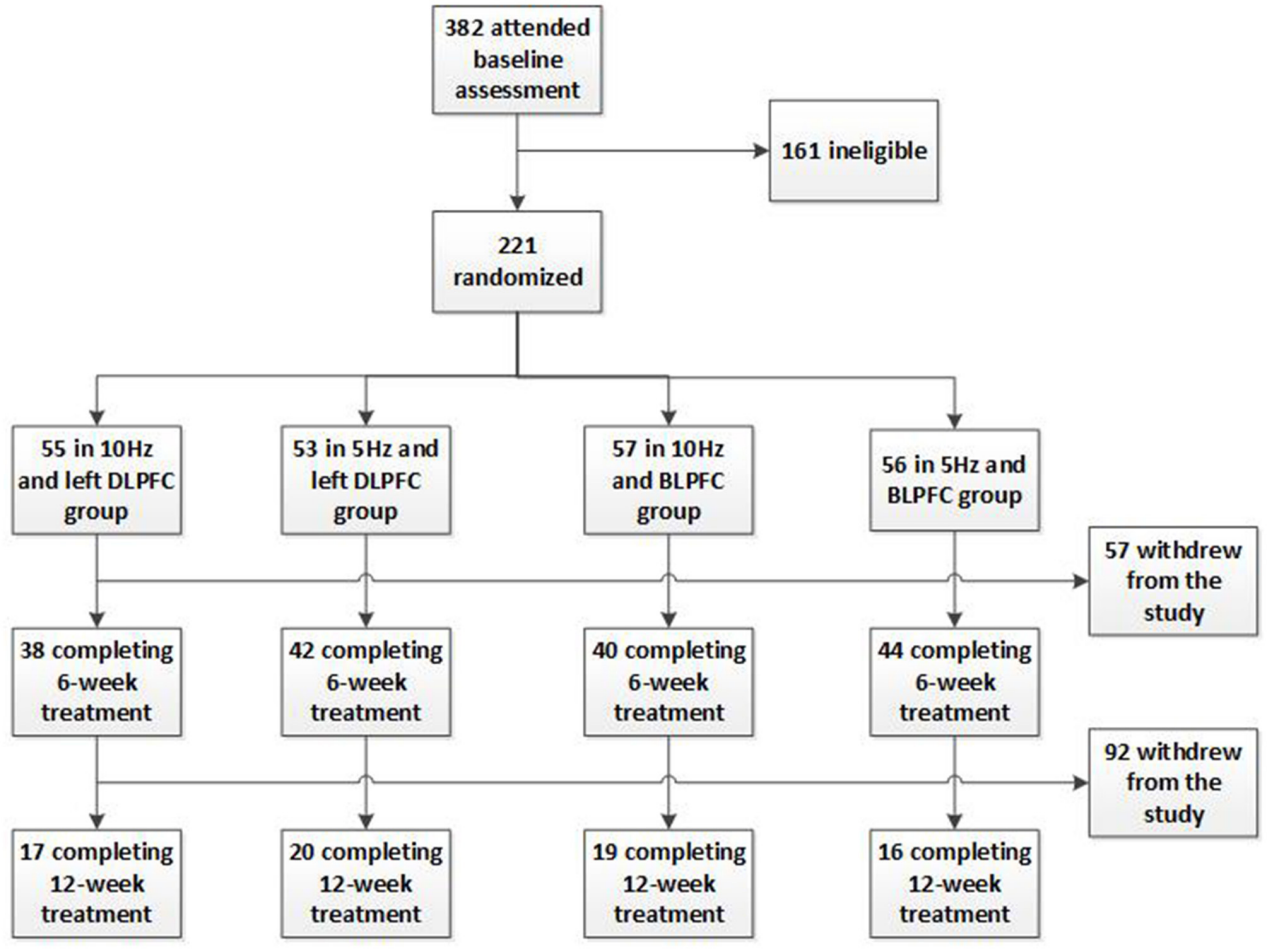
Flowchart of subjects through the study.

A total of 221 participants were enrolled and randomly assigned into four parallel rTMS treatment groups. There were 55 participants in the 10 Hz and left DLPFC group, 53 participants in the 5 Hz and left DLPFC group, 57 participants in the 10 Hz and bilateral DLPFC group, and 56 participants in the 5 Hz and bilateral DLPFC group. Figure 1 shows the disposition of the participants. The groups were well-balanced and comparable in age, gender, ethnicity, marital status, education level, HDRS score at baseline, disease course (the months from the first episode to the time at the interview), and recurrent times (including the current episode) (Table 1).

**Table 1:** Socio-demographic and clinical characteristics of the sample (*N* = 221).

**Variable**	**Figure-8 coil in left DLPFC**		**Round coil in bilateral DLPFC**		***F/*χ^2^**	***P***
	**10 Hz(*n* = 55)**	**5 Hz(*n* = 53)**	**10 Hz(*n* = 57)**	**5 Hz(*n* = 56)**		
Age (mean±SD)	37.6 ± 12.2	34.9 ± 11.0	38.5 ± 14.2	38.9 ± 13.3	1.01	0.39
Gender					5.78	0.12
Male	22	28	20	30		
Female	33	25	37	26		
Ethnicity					4.93	0.17
Han	50	51	51	55		
Others	5	2	6	1		
Religious belief					1.40	0.71
No	51	47	50	52		
Yes	4	6	7	4		
Marital status					5.10	0.53
Never married	17	20	19	23		
Current married	34	27	28	28		
Divorce or widowed	4	6	10	5		
Education level					4.39	0.62
Middle school or below	16	14	19	20		
Bachelor	32	29	28	31		
Master or above	7	10	11	5		
Family month income per person (RMB)					11.03	0.09
0–1,999	12	12	20	24		
2,000–4,999	23	27	21	15		
5,000 and above	20	14	16	17		
HDRS score (mean±SD)	29.9 ± 5.2	30.3 ± 5.7	30.3 ± 5.4	29.5 ± 5.1	0.25	0.86
Disease course (months) (mean±SD)	60.3 ± 77.6	63.9 ± 64.9	47.8 ± 62.8	54.6 ± 81.7	3.43	0.33
Recurrent times (mean±SD)	0.8 ± 1.5	0.6 ± 1.6	0.6 ± 1.2	0.6 ± 1.0	1.68	0.64

### Primary Outcome Variables

#### Comparing Percent Change of HDRS Score at Week 6 and Week 12 to Baseline

HDRS scores significantly decreased from pre-treatment to post-treatment. It showed that the curve of HDRS scores on a weekly interval in the first 6 weeks declined quickly, while 6–12 weeks dropped smoothly. Of the 221 participants, the HDRS score at 6 and 12 weeks remarkably decreased 56.5 and 59.7%, respectively, compared to the baseline. The HDRS score decreases of each group are displayed in Table 2.

**Table 2:** The decrease of HDRS score and CGI score at week 6 and week 12.

**Variable**	**Figure-8 coil in left DLPFC**		**Round coil in bilateral DLPFC**	
	**10 Hz (*n* = 55)**	**5 Hz(*n* = 53)**	**10 Hz(*n* = 57)**	**5 Hz(*n* = 56)**
HDRS at week 6	16.9 ± 7.3	16.9 ± 6.7	17.1 ± 8.1	17.3 ± 7.5
HDRS at week 12	18.0 ± 8.0	17.8 ± 7.3	17.7 ± 8.9	18.4 ± 7.9
CGI at week 6	2.4 ± 1.3	2.6 ± 1.2	2.3 ± 1.5	2.2 ± 1.3
CGI at week 12	2.4 ± 1.4	2.6 ± 1.3	2.4 ± 1.5	2.3 ± 1.3

#### Comparing the HDRS Scores Among the Treatment Groups Over Time

All of the participants were analyzed by ITT. The ANOVAs of HDRS scores up to 6 and 12 weeks with repeated measure of time both showed a significant effect of duration (*F* = 472.18, *P* < 0.01 and *F* = 414.15, *P* < 0.01). For four treatment groups, however, there were no statistical differences (Table 3). There was also no statistical significance when time was interacted with the treatment group. The same results were obtained when the data were analyzed by PP analysis. HDRS scores of four treatment groups at each week are shown in Supplementary Table 1.

**Table 3:** The results of repeated measure of ANOVA of HDRS and CGI score by ITT.

**Duration**	**Coil and stimulating location**		**Frequency**		**Coil and stimulating location × Frequency**		**Time**		**Time × Coil and stimulating location**		**Time × Frequency**		**Time × Coil and stimulating location × Frequency**	
	***F***	***P***	***F***	***P***	***F***	***P***	***F***	***P***	***F***	***P***	***F***	***P***	***F***	***P***
HDRS at week 6	0.13	0.72	0.53	0.47	0.35	0.56	472.18	<0.01	0.80	0.51	0.42	0.77	0.52	0.70
HDRS at week 12	0.12	0.73	0.51	0.47	0.60	0.44	414.15	<0.01	0.58	0.70	0.36	0.86	0.78	0.55
CGI at week 6	0.23	0.63	1.06	0.31	0.64	0.43	348.47	<0.01	1.49	0.22	0.31	0.80	1.17	0.32
CGI at week 12	0.18	0.67	1.07	0.30	0.62	0.43	296.61	<0.01	1.05	0.38	0.32	0.86	0.93	0.44

### Secondary Outcome Variables

CGI scores also significantly decreased compared with the baseline. Of the 221 participants, the CGI score at 6 and 12 weeks decreased 48.3 and 49.8%, respectively, compared to the baseline. Similar to the HDRS scores, there were no significant differences in CGI scores from an ANOVA comparing the four treatment groups. The decreased scores of each group are displayed in Table 2. For the repeated ANOVA of CGI score, similar results were found with the HDRS score, with significant duration effects and without differences among four treatment groups (Table 3).

### Response Analysis

The total response rates of treatment at the end of the 6th week and the 12th week were 63.8% (95% CI: 57.4–70.1%) and 67.4% (95% CI: 61.2–73.6%), respectively. There was no statistical difference in response rates, using Chi-squared analysis across the four treatment groups at Week 6 and Week 12. GEE analysis on the influence of duration and treatment parameters only found duration of treatment as a statistically significant predictor (*P* < 0.01). When weekly response rates were compared with previous weeks, only the rates up until Week 5 were significantly different when compared with the previous week's rates, suggesting that response after Week 5 was stable (Table 4).

**Table 4:** Test of within-participants contrast of response rate.

**Contrast**	**Contrast**	**Standard**	**Wald**	***P***
	**estimate**	**error**	**chi-square**	
Week 1 vs. Week 2	−0.14	0.03	17.18	<0.01
Week 2 vs. Week 3	−0.08	0.04	4.63	0.03
Week 3 vs. Week 4	−0.09	0.03	7.21	0.01
Week 4 vs. Week 5	−0.08	0.03	6.39	0.01
Week 5 vs. Week 6	−0.04	0.03	1.67	0.20
Week 6 vs. Week 7	−0.02	0.05	0.20	0.65
Week 7 vs. Week 8	<0.01	0.05	0.01	0.94
Week 8 vs. Week 9	−0.08	0.04	5.86	0.02
Week 9 vs. Week 10	−0.01	0.03	0.13	0.72
Week 10 vs. Week 11	0.01	0.04	0.03	0.86
Week 11 vs. Week 12	<0.01	0.04	0.01	0.98

### Analysis of Symptom Clusters

The HDRS score can be divided into six symptom clusters, including anxiety/somatization, weight, cognition, slowing, insomnia, and atypical. In order to understand the influence of rTMS on six aspects of MDD, each symptom cluster was analyzed separately. The repeated measure ANOVA with ITT analysis revealing all of the symptom clusters showed that course of treatment, up to 6 and 12 weeks, was a significance predictor. There was no significant difference in the symptom clusters among four treatment groups (Table 5).

**Table 5:** The results of repeated measure of ANOVA of the Cclusters of HDRS by ITT.

**Duration**	**Cluster of HDRS**	**Coil and stimulating location**		**Frequency**		**Coil and stimulating location × Frequency**		**Time**		**Time × Coil and stimulating location**		**Time × Frequency**		**Time × Coil and stimulating location × Frequency**	
		***F***	***P***	***F***	***P***	***F***	***P***	***F***	***P***	***F***	***P***	***F***	***P***	***F***	***P***
Week 6	Anxiety/somatization	0.27	0.61	3.03	0.08	<0.01	0.99	183.66	<0.01	0.10	0.98	0.22	0.92	0.85	0.49
	Weight	0.14	0.71	0.96	0.33	0.36	0.55	42.44	<0.01	0.67	0.56	2.02	0.11	0.59	0.61
	Cognition	0.39	0.54	0.56	0.45	0.14	0.71	123.64	<0.01	0.70	0.58	1.41	0.23	0.20	0.93
	Slowing	1.66	0.20	<0.01	0.97	1.97	0.16	257.85	<0.01	1.56	0.19	0.22	0.92	0.71	0.58
	Insomnia	7.45	<0.01	0.77	0.38	0.26	0.61	127.98	<0.01	0.70	0.60	0.91	0.46	1.58	0.17
	Atypical	0.21	0.65	0.03	0.87	0.12	0.73	233.12	<0.01	0.52	0.70	0.24	0.90	1.22	0.30
Week 12	Anxiety/somatization	0.15	0.70	3.55	0.06	0.50	0.48	164.88	<0.01	0.20	0.97	0.48	0.80	2.03	0.07
	Weight	0.23	0.63	0.67	0.42	0.78	0.38	40.30	<0.01	0.64	0.61	1.70	0.16	0.43	0.76
	Cognition	0.88	0.35	0.70	0.41	0.04	0.84	97.57	<0.01	0.72	0.62	0.93	0.47	0.38	0.88
	Slowing	1.05	0.31	<0.01	0.99	1.11	0.29	232.67	<0.01	1.22	0.30	0.19	0.96	1.06	0.38
	Insomnia	7.56	<0.01	1.08	0.30	0.02	0.88	112.85	<0.01	0.55	0.75	0.77	0.58	1.41	0.21
	Atypical	0.20	0.66	<0.01	0.95	0.19	0.67	206.21	<0.01	0.38	0.84	0.21	0.94	0.89	0.48

### Dropout

Due to the long duration of treatment, dropout occurred in each treatment group. The results of a Chi-square test of the distribution of complete cases and dropouts among the four treatment groups at Week 6 and Week 12 conveyed that there was no statistical difference among the four groups (Supplementary Table 2). Considering that the dropout rates after 6 weeks seemed to be high, the randomness of dropout needed to be confirmed. The repeated measure ANOVA of HDRS scores was applied between the dropout group and completion group for each week after Week 6 using PP data and results revealed no statistical difference of HDRS scores between the two groups (Supplementary Table 3). It was demonstrated that the treatment effects of dropouts were not different from that of the completion group.

### Side Effects

In general, rTMS was well-tolerated and there were no serious side effects that occurred in the treatment process. The main types of side effects the participants reported included bodily pain, such as headache, toothache, dizziness, and numbness in the scalp. Most of these side effects were mild and temporary. No seizures occurred in any treatment group.

## Discussion

To our knowledge, this is the first randomized controlled rTMS trial that simultaneously compared two stimulation sites of left DLPFC with figure-eight coil vs. bilateral DLPFC with round coil and two frequencies of 5 vs. 10 Hz, with such a large sample size in 6-week duration of prolonged treatment, and a 6-week optional treatment. In order to compare the efficacy of the parameters of rTMS treatment for MDD, four active rTMS conditions with two stimulation frequencies and two sites were evaluated at the same time. Remarkably decreased HDRS scores indicated a statistically significant effect of treatment duration, which is consistent with previous research (6, 12, 13, 31). However, the study failed to find a better parameter. In general, rTMS treatments were well-tolerated without any serious adverse events.

In 2008, the U.S. FDA cleared rTMS treatment for MDD with the protocol of 10 Hz in left DLPFC as the effective parameter of rTMS to treat MDD. Considering the results of equivalent effect in two locations with two frequencies, this study suggests that the other parameters in the trial have a compatible effect on MDD to the specific parameters proven by many previous studies worldwide. Actually, some studies that compared the effect difference of stimulation parameters can support this point (15, 32, 33). A small sample study conducted by Shajahan et al. (34) in 2002 explored the effect differences of 5, 10, and 20 Hz, which produced consistent results with this trial with no statistical difference among different frequencies. Another recent study also compared the efficacy difference of 1 Hz in right DLPFC and 10 Hz in left DLPFC, and similarly failed to find a significant difference between two groups (24). However, the results of previous studies were mostly yielded from a small sample size and consequently had low statistical power to support the findings. As for the mechanism of the negative results of frequencies, the individualization of cortical oscillators might be an explanation. One hypothesis was that it might improve the effectiveness of rTMS treatment by synchronizing the rTMS pulse to the patient's own frequency, which is called synchronous TMS (sTMS) (35). Possibly, the protocols employed in the current study have not shown significant difference as they were not synchronized to the patient's rhythm. However, whether the sTMS could improve the effect remains to be proven. It should be noted that bilateral DLPFC in the current study (10 or 5 Hz on both sides) was different from that in previous studies (low frequency on the right side and high frequency on the left side). However, both of the bilateral protocols failed to find differences with left DLPFC. It was suggested that adding right- to left-sided treatment did not enhance efficacy. Left DLPFC was the conventional target for stimulation in MDD because of the significance in mood regulation. However, mood is regulated by a network of brain regions (including the pre-frontal, parietal, and other regions) and focal lesions could lead to mood disturbance (36). A similar effect of rTMS in left DLPFC and bilateral DLPFC could be partially illustrated by this point. The results of this trial and other similar researches hence imply that the protocols should not be only limited to 10 Hz in left DLPFC. This result could be a supplementary for the treatment parameter of MDD. Although there was no serious side effect occurring in the trial, the potential for seizure induction with rTMS is related to the increase in frequency (23). There was also evidence that low frequency may have some anticonvulsant effects (37). In this aspect, low frequency may be much safer than high frequency. The comparable results of left and bilateral DLPFC also raised questions about the pathogenesis of MDD. The role of different brain regions for MDD still needs further research.

The findings in the current trial also suggested no additional effect when the treatment was prolonged. The response rate plateaued and had no statistical difference after 5 weeks. Similar to drug therapy, the effect of rTMS came into consolidation with the maintenance period after the quick relief of MDD. Only a few studies have been able to provide insight into the effectiveness of treatment duration. Heretofore, no rTMS trial lasted 12 weeks. Some studies that lasted between 4 and 6 weeks demonstrated that the therapeutic effects still improved in the 4th or 6th week, but parts of the research was not a controlled trial (38–40).

Recent research conveyed that the rTMS had a distinct effect on sleeping disorders (11, 41, 42). A study conducted by George in 2000 indicated that rTMS had a more robust influence on insomnia than other symptom clusters (11). The current study found a significant effect of figure-eight coil over left DLPFC on insomnia compared to round coil over bilateral DLPFC, with a negative effect of interaction with time. The results prompted that the MDD patients with severe insomnia problem may use rTMS to improve their symptoms, and different parameters might have different effects, which needed further research.

A 56.5% HDRS score decrease and 67.4% response rate produced greater improvement from rTMS treatment than that documented in the current literature. It had to be admitted that the high response included placebo effect inevitably. Excluding the reason that dropouts were mostly non-responders, which was proven invalid above, a potential explanation may be the washout of antidepressant and anxiolytics. The participants were asked to wash out the current medications, which could make the condition worse in some cases. They would thus more easily be responders to rTMS, resulting in better efficacy of rTMS treatment.

There were some limits of the study worthy of consideration. First, we did not set a placebo arm in the trial. Therefore, it could not be ruled out that some non-specific effects may improve the effect of rTMS. However, based on studies on rTMS for treating MDD in the recent 20 years, it is clearly proven that the efficacy of rTMS for MDD is much higher than that of placebo (7, 11, 43). Also, as all of the participants were requested to be free of any antidepressant and benzodiazepines during the study period, it is unlikely and unethical to set a sham-control group with MDD patients without medicine and any other therapy for 12 weeks. Secondly, the rate of dropout seemed to be high in the trial. Given the time commitment and the long duration of therapy, it was difficult to maintain full compliance, which brought some challenges regarding the validity of the findings. Therefore, we evaluated the different feature of the dropout. Among the four treatment groups, the dropout rates appeared to be symmetrical. Also, the trend of HDRS scores between the dropout group and the completion group had no statistical difference. Meanwhile, the ITT analysis and PP analysis yielded similar results when evaluating the efficacy of different parameters of the rTMS treatment for MDD. Therefore, the dropout occurred randomly among four treatment groups, and the results of intergroup comparison could not be influenced by the dropout. Some researchers may suspect that the findings are difficult to translate into patient care due to the high dropouts. However, most of the dropouts emerged after the 6th week, and we have declared that the efficacy plateaued after a 5-week course of treatment. Although we recommend a prolonged treatment, it could be inferred that a shortened duration, at least a 5-week course of treatment, is feasible in clinical practice. Thirdly, the number of stimuli might be another positive treatment parameter. The number of stimuli of the current research (1,200 stimuli per session in the 10-Hz groups and 600 stimuli in the 5-Hz groups) was different from several previous studies, which might also limit the generalization of the study. However, the current results might oppose the notion that more pulses were needed because 5 and 10 Hz had similar results, which needed further research.

In conclusion, this study adds to the growing literature studying the effects among different protocols. It provides confirmatory evidence that different treatment parameters of rTMS can be considered for MDD. The study yielded comparable results among different parameters. Considering the smaller possibility of side effect with lower frequency, 5 Hz might be more recommended compared to 10 Hz. The present data also document that the response in the first 5 weeks is significant and then becomes stable. However, concerns continue to be raised as to the mechanism behind the effects and whether the effects of rTMS are applicable to practice; therefore, further research on MRI and other aspects are still needed.

## Data Availability Statement

The raw data supporting the conclusions of this article will be made available by the authors, without undue reservation.

## Ethics Statement

The studies involving human participants were reviewed and approved by the Ethical Committee of the Sixth Hospital of Peking University. The patients/participants provided their written informed consent to participate in this study.

## Author Contributions

YH originally designed the idea of the study and has been responsible for obtaining funding. YH, YJ, and ZL contributed to the study design and development of study instruments. XM collected the data and did the primary data analysis. TZ undertook data cleaning, checking and coding, did the analysis for the study, and wrote the initial draft. YH and YJ contributed to amendment of the manuscript, suggestions for data analysis, conceived the idea for this paper, supervised and checked the analysis, and wrote the final manuscript. All authors contributed to the interpretation of data and the approval of the final report.

## Conflict of Interest

The authors declare that the research was conducted in the absence of any commercial or financial relationships that could be construed as a potential conflict of interest.
