# Supplementary material for: Treatment for Major Depressive Disorder by Repetitive Transcranial Magnetic Stimulation in Different Parameters: A Randomized Double-Blinded Controlled Trial
Source: Front Psychiatry. 2021 Apr 6;12:623765. doi: 10.3389/fpsyt.2021.623765 (PMC8055955; doi:10.3389/fpsyt.2021.623765)
Supplement: Supplementary file 1 [file Data_Sheet_1.docx]

Table 1 HDRS Scores of Four Treatment Groups

| Duration | Figure-8 coil in left DLPFC | |  | Round coil in BLPFC | | *F* | *P* |
| --- | --- | --- | --- | --- | --- | --- | --- |
|  | 10 Hz（N=55） | 5 Hz（N=53） |  | 10 Hz（N=57） | 5 Hz（N=56） |  |  |
| Week 1 | 29.93±5.22 | 30.25±5.67 |  | 30.25±5.42 | 29.48±5.06 | 0.44 | 0.73 |
| Week 2 | 19.67±7.79 | 18.42±7.10 |  | 19.81±7.04 | 19.18±6.07 | 0.53 | 0.66 |
| Week 3 | 16.25±6.77 | 16.84±5.83 |  | 15.78±6.46 | 15.39±5.38 | 0.31 | 0.82 |
| Week 4 | 14.07±6.44 | 14.84±6.64 |  | 14.78±5.96 | 15.27±5.98 | 0.08 | 0.97 |
| Week 5 | 13.58±6.87 | 13.47±5.91 |  | 13.93±6.22 | 13.30±6.30 | 0.12 | 0.95 |
| Week 6 | 12.29±7.20 | 12.21±6.04 |  | 12.20±5.77 | 11.60±5.81 | 0.24 | 0.87 |
| Week 7 | 11.82±6.13 | 12.24±6.05 |  | 11.40±5.73 | 11.27±5.11 | 1.22 | 0.31 |
| Week 8 | 13.56±6.84 | 10.90±6.32 |  | 11.45±5.37 | 10.87±5.10 | 0.33 | 0.81 |
| Week 9 | 11.45±5.39 | 11.62±6.30 |  | 11.64±6.17 | 10.26±6.02 | 2.02 | 0.12 |
| Week 10 | 11.09±6.15 | 11.08±7.17 |  | 12.48±6.15 | 8.08±5.36 | 1.19 | 0.32 |
| Week 11 | 12.00±5.39 | 10.82±6.07 |  | 11.45±6.56 | 8.58±5.90 | 0.38 | 0.77 |
| Week 12 | 11.06±6.68 | 11.05±7.26 |  | 10.89±7.53 | 9.05±5.98 | 0.54 | 0.66 |

Table 2 Dropout among Four Groups after Week 6

| Week of dropout | Figure-8 coil in left DLPFC | |  | Round coil in BLPFC | | χ*^2^* | *P* |
| --- | --- | --- | --- | --- | --- | --- | --- |
|  | 10 Hz（N=55） | 5 Hz（N=53） |  | 10 Hz（N=57） | 5 Hz（N=56） |  |  |
| Week 6 | 17 | 11 |  | 17 | 12 | 2.50 | 0.48 |
| Week 7 | 30 | 22 |  | 28 | 26 | 1.92 | 0.59 |
| Week 8 | 33 | 24 |  | 32 | 29 | 2.60 | 0.46 |
| Week 9 | 33 | 29 |  | 36 | 32 | 0.91 | 0.82 |
| Week 10 | 36 | 31 |  | 37 | 37 | 0.87 | 0.83 |
| Week 11 | 38 | 31 |  | 38 | 37 | 1.49 | 0.69 |
| Week 12 | 38 | 33 |  | 38 | 40 | 1.14 | 0.77 |

Table 3 The Results of Repeated ANOVA of HDRS between

Dropout Group and Completion Group

| Week of Dropout | Mean Square | *F* | *P* |
| --- | --- | --- | --- |
| Week 7 | 178.37 | 1.11 | 0.29 |
| Week 8 | 14.30 | 0.08 | 0.78 |
| Week 9 | 0.06 | <0.01 | 0.99 |
| Week 10 | 203.16 | 0.86 | 0.36 |
| Week 11 | 63.44 | 0.25 | 0.62 |
| Week 12 | 126.16 | 0.43 | 0.52 |
